# Effects of Vitamin D_2_ (Ergocalciferol) and D_3_ (Cholecalciferol) on Atlantic Salmon (*Salmo salar*) Primary Macrophage Immune Response to *Aeromonas salmonicida* subsp. *salmonicida* Infection

**DOI:** 10.3389/fimmu.2019.03011

**Published:** 2020-01-14

**Authors:** Manuel Soto-Dávila, Katherinne Valderrama, Sabrina M. Inkpen, Jennifer R. Hall, Matthew L. Rise, Javier Santander

**Affiliations:** ^1^Marine Microbial Pathogenesis and Vaccinology Lab, Department of Ocean Sciences, Memorial University of Newfoundland, St. John's, NL, Canada; ^2^Department of Ocean Sciences, Memorial University of Newfoundland, Ocean Science Centre, St. John's, NL, Canada; ^3^Aquatic Research Cluster, CREAIT Network, Ocean Sciences Centre, Memorial University of Newfoundland, St. John's, NL, Canada

**Keywords:** Atlantic salmon, vitamin D_3_, vitamin D_2_, innate immunity, primary macrophages, *Aeromonas salmonicida*, Gram-negative

## Abstract

Vitamin D_2_ (ergocalciferol) and vitamin D_3_ (cholecalciferol) are fat-soluble secosteroid hormones obtained from plant and animal sources, respectively. Fish incorporates vitamin D_2_ and D_3_ through the diet. In mammals, vitamin D forms are involved in mineral metabolism, cell growth, tissue differentiation, and antibacterial immune response. Vitamin D is an essential nutrient in aquafeeds for finfish. However, the influence of vitamin D on fish cell immunity has not yet been explored. Here, we examined the effects of vitamin D_2_ and vitamin D_3_ on *Salmo salar* primary macrophage immune response to *A. salmonicida* subspecies *salmonicida* infection under *in vitro* conditions. We determined that high concentrations of vitamin D_2_ (100,000 ng/ml) and D_3_ (10,000 ng/ml) affect the growth of *A. salmonicida* and decrease the viability of *S. salar* primary macrophages. In addition, we determined that primary macrophages pre-treated with a biologically relevant concentration of vitamin D_3_ for 24 h showed a decrease of *A. salmonicida* infection. In contrast, vitamin D_2_ did not influence the antibacterial activity of the *S. salar* macrophages infected with *A. salmonicida*. Vitamin D_2_ and D_3_ did not influence the expression of canonical genes related to innate immune response. On the other hand, we found that *A. salmonicida* up-regulated the expression of several canonical genes and suppressed the expression of *leukocyte-derived chemotaxin 2* (*lect-2*) gene, involved in neutrophil recruitment. Primary macrophages pre-treated for 24 h with vitamin D_3_ counteracted this immune suppression and up-regulated the transcription of *lect-2*. Our results suggest that vitamin D_3_ affects *A. salmonicida* attachment to the *S. salar* primary macrophages, and as a consequence, the *A. salmonicida* invasion decreased. Moreover, our study shows that the positive effects of vitamin D_3_ on fish cell immunity seem to be related to the *lect-2* innate immunity mechanisms. We did not identify positive effects of vitamin D_2_ on fish cell immunity. In conclusion, we determined that the inactive form of vitamin D_3_, cholecalciferol, induced anti-bacterial innate immunity pathways in Atlantic salmon primary macrophages, suggesting that its utilization as a component of a healthy aquafeed diet in Atlantic salmon could enhance the immune response against *A. salmonicida*.

## Introduction

Vitamin D is a fat-soluble secosteroid hormone that plays a crucial role in calcium and phosphorus homeostasis, cardiovascular physiology, cell proliferation and differentiation, among other functions (1–6). In fish, vitamin D is involved in the endocrine control of calcium and phosphorus homeostasis, similar to mammals (4). Also, it has been shown that vitamin D can act as an immunomodulatory agent in mammals (7, 8).

In contrast to terrestrial vertebrates, fish are not able to obtain vitamin D through the photochemical pathway, thus fish must ingest vitamin D from dietary sources (9). In freshwater and marine environments, the main dietary sources of vitamin D are phytoplankton and zooplankton (9). Phytoplankton provide fish with vitamin D_2_ (ergocalciferol), while zooplankton provide them with vitamin D_3_ (cholecalciferol) (10).

The beneficial stimulatory effects of vitamins D_2_ and D_3_ on innate immunity have been described in humans and other mammals (5, 8, 11–14). The positive effects of vitamins in fish are well-established, and currently vitamins D_2_ and D_3_ are essential components of aquafeed diets (4). Additionally, vitamin D is utilized as an adjuvant in aqua-vaccine preparations (15). However, the role of vitamin D in fish physiology is still enigmatic, and the immune stimulant mechanisms against infectious diseases are unknown.

Atlantic salmon (*Salmo salar*) is a high-value cultured finfish species, and the main species cultured in Canada, Chile, UK, and Norway (16–18). Infectious diseases caused by bacterial pathogens such as *Renibacterium salmoninarum, Piscirickettsia salmonis, Vibrio anguillarum*, and *Aeromonas salmonicida* subsp. *salmonicida* (19–24), have affected this industry since its origin (25, 26). Currently, several measures are utilized to prevent infectious diseases in the Atlantic salmon aquaculture industry, including a healthy diet that includes immunostimulants (27–29).

Functional constituents of healthy aquafeed diets like essential nutrients (such as vitamins, probiotics, prebiotics, and immunostimulants) are currently being considered to improve not only fish growth and stress tolerance, but also the resistance to diseases by enhancing non-specific defense mechanisms (30, 31). These essential nutrients are capable of directly activating immune mechanisms, such as phagocytic activity (e.g., macrophages and neutrophils), complement system, lysozyme activity, and others (32–34). Phagocytosis is an active host defense mechanism, involving the action of monocytes, dendritic cells, neutrophils, and macrophages (34–36). From these phagocytic leukocytes, the macrophages play an important role linking the innate and adaptive immune responses, and previous studies have shown that immunostimulants (i.e., fructooligosaccharides, mannanoligosaccharides) can successfully enhance the phagocytic activity in rainbow trout and European sea bass (34, 37, 38).

Immunostimulants are natural compounds that have been shown to be safe and effective for fish (30, 33, 39). In humans, vitamin D plays an important role in the suppression of pro-inflammatory cytokines such as *il-17, il-1b*, and *tnf-*α in individuals affected by type 2 diabetes and autoimmune diseases, preventing chronic inflammation (40, 41). Additionally, vitamin D significantly reduces *Staphylococcus aureus* infection in pre-treated bovine epithelial cells and modulates the expression of innate immune related genes (8, 13, 14). These studies suggest that vitamin D could trigger similar protective effects in fish. Here, we evaluate the effects of vitamins D_2_ and D_3_ on the innate immune responses of Atlantic salmon primary macrophages to *A. salmonicida* infection.

## Materials and Methods

### *Aeromonas salmonicida* Growth Conditions

*A. salmonicida* was grown in accordance to the protocol used by Soto-Dávila et al. (42). Briefly, a single colony of *A. salmonicida* J223 (24) was incubated in 3 ml of Trypticase Soy Broth (TSB, Difco, Franklin Lakes, NJ) at 15°C in a 16 mm diameter glass tube and placed in a roller for 24 h. After growth, 300 μl of the overnight culture was added to 30 ml of TSB media using a 250 ml flask and incubated for 24 h at 15°C with aeration (180 rpm). After bacteria reached an O.D. 600 nm ~0.7 (1 × 10^8^ CFU ml^−1^), the bacterial culture was centrifuged at 6,000 rpm at room temperature for 10 min. The pellet was washed twice with phosphate buffered saline [PBS; 136 mM NaCl, 2.7 mM KCl, 10.1 mM Na_2_HPO_4_, 1.5 mM KH_2_PO_4_ (pH 7.2)] and centrifuged at 6,000 rpm at room temperature for 5 min, and finally resuspended in 300 μl of PBS (~5 × 10^10^ CFU ml^−1^). The concentrated bacterial inoculum was serially diluted and quantified by plating onto TSA supplemented with Congo red (50 μg ml^−1^) for 4 days.

### Vitamin D_2_ and D_3_ Inhibitory Effects on *A. salmonicida* Growth

To determine whether vitamin D_2_ or vitamin D_3_ have inhibitory effect on *A. salmonicida* growth, 30 μl of the overnight growth bacteria were placed in 3 ml of TBS containing different concentrations of vitamin D_2_ (10; 100; 1,000; 10,000; and 100,000 ng ml^−1^; Sigma-Aldrich) or vitamin D_3_ (10; 100; 1,000; and 10,000 ng ml^−1^; Sigma-Aldrich). Bacterial growth was measured by O.D. 600 nm until 48 h. The effect of each vitamin concentration on *A. salmonicida* growth was measured in triplicate, and TSB media containing the respective vitamin D concentration was utilized as a blank.

### Fish Holding

Adult specimens of Atlantic salmon 4.0 ± 0.1 kg (mean ± SE) were obtained from the Dr. Joe Brown Aquatic Research Building (JBARB) at the Department of Ocean Sciences, Memorial University of Newfoundland, Canada. The animals were kept in 37 m3 tanks, with flow-through (100 l min^−1^) seawater (6.5°C) and ambient photoperiod. The individuals were fed twice per day with commercial salmonid dry pellets (Skretting Optiline Microbalance 3000 EP, 12.0 mm pellets: 38% protein, 33% fat, 1.6% calcium, 1.5% fiber, 1% phosphorus) with a ration of 0.5% of body weight per day. The experiment was performed in accordance with the guidelines of the Canadian Council on Animal Care and approved by Memorial University of Newfoundland's Institutional Animal Care Committee (protocols #17-01-JS; #17-02-JS).

### Macrophage Isolation

Primary macrophages were isolated from Atlantic salmon head kidney. Tissues from 6 fish were aseptically removed and individually minced through 100 μm nylon sterile cell strainers (Fisher Scientific, Thermo Fisher Scientific, Waltham, MA, USA) in isolation media [(Leibovitz-15 (Gibco®, Gran Island, NY, USA) supplemented with 250 μg ml^−1^ heparin, 100 U ml^−1^ penicillin, 100 μg ml^−1^ streptomycin, and 0.1% Fetal Bovine Serum (FBS)]. After this period, 4 ml of cell suspension were centrifuged (1,000 × g at 4°C) for 30 min in a 34/51% Percoll gradient (GE Healthcare, Uppsala, Sweden). Macrophages collected from the macrophage-enriched interface were washed with PBS twice and the number and viable cells were determined using the Countness™ cell counter (Invitrogen), and trypan blue stain (Invitrogen). After determining the cell concentration (number of cells per ml^−1^) of each sample, the primary macrophages were seeded in 22 mm 12-well or 35 mm 6-well cell-culture multidishes (Thermo Scientific, Roskilde, Denmark) at a concentration of 1 x 10^7^ cells ml^−1^. The plates were incubated at 15°C for 24 h in isolation media. After this period the cells were washed with PBS and incubated at 15°C for an additional 4 days in 1 ml of culture media (Leibovitz-15 (Gibco®), supplemented with 0.1% 2-Mercaptoethanol, 100 U ml^−1^ penicillin, 100 μg ml^−1^ streptomycin, and 5% FBS) to allow cell attachment until the assays were performed.

### Vitamin D_2_ and D_3_ Toxicity in Atlantic Salmon Primary Macrophages

Atlantic salmon primary macrophages were seeded in 12-well cell-culture multidishes at a concentration of 1 x 10^7^ cells ml^−1^. After 4 days of incubation the culture media was removed, cells washed with PBS, and 1 ml of culture media containing different concentrations of vitamin D_2_ (10; 100; 1,000; 10,000; and 100,000 ng ml^−1^) or vitamin D_3_ (10; 100; 1,000; and 10,000 ng ml^−1^) was added. Twenty-four hours and 48 h post-vitamin treatment, cells were treated with 500 μl of trypsin-EDTA (0.5%; Gibco) for 10 min, and then trypsin was inactivated with 500 μl of culture media. The cells were stained with trypan blue (0.4%; Invitrogen) in a ratio of 1:1 (10 μl: 10 μl) and quantified using Countess™ Cell Counting Chamber Slides (Invitrogen) and Countess® Automated Cell Counter (Invitrogen) according to the manufacturer's instructions. Viability of cells was determined for each vitamin D_2_ and D_3_ concentration and the control group. All samples were taken from 6 individual fish.

### Gentamicin Exclusion Assay

Infections of primary macrophages with *A. salmonicida* were performed according to the protocol used by Soto-Dávila et al. (42) with modifications. Briefly, after 4 days, the primary macrophages were washed with 1 ml of PBS and inoculated with 1 ml of culture media without antibiotics containing either 100 ng ml^−1^ of vitamin D_2_ or D_3_ for 24 h. After this period, media was removed, cells washed with 1 ml of PBS, and pre-treated primary macrophage monolayers were infected with 10 μl of bacterial suspension [~1 × 10^7^ cells ml^−1^; Multiplicity of Infection (MOI) 1:1 (bacteria:macrophage)] and incubated at 15°C. For attachment, after 1 h of infection, the infected primary macrophage monolayers were washed twice with 1 ml of PBS and then lysed using 400 μl of Triton X100 (0.01%; Sigma) for 10 min (43). After this 600 μl of PBS was added to complete 1 ml of lysed macrophage suspension. Then, the lysed macrophage suspensions were serially diluted (1:10) and plate/counted on TSA plates supplemented with Congo red to determine the number of viable *A. salmonicida* per monolayer. The plates were incubated at 15°C for 5 days to determine the CFU per well.

For invasion, the primary macrophages were infected for 1 h, washed twice with 1 ml of PBS, and 1 ml of fresh culture media supplemented with gentamicin (10 μg ml^−1^, a higher concentration than the minimal inhibitory concentration) (24) was added. Gentamicin treatment was applied to kill remaining extracellular bacteria. After 2, 3, and 4 h of infection, the infected primary macrophage monolayers were washed twice with PBS and then lysed using 400 μl of Triton X100 (0.01%) for 10 min (43). After this, 600 μl of PBS was added to complete 1 ml of lysed macrophage suspension. Then, the lysed macrophage suspensions were serially diluted (1:10) and plate/counted on TSA plates supplemented with Congo red to determine the number of viable intracellular *A. salmonicida* per monolayer. The plates were incubated at 15°C for 5 days to determine the CFU per well. All samples were taken from 6 individual fish.

### Vitamin D_2_ and D_3_ Pre-treated Primary Macrophage Viability After *A. salmonicida* Infection

To determine the viability of infected primary macrophages, the cells were seeded in 12 wells plates, pre-treated with 100 ng ml^−1^ of vitamin D_2_ or vitamin D_3_, infected with *A. salmonicida*, and processed following the method used during the gentamicin exclusion assay. Cells were washed with 1 ml of PBS and then treated with 500 μl of trypsin-EDTA (0.5%; Gibco) for 10 min. After this period, the trypsin was inactivated with 500 μl of culture media. The primary macrophages were stained using trypan blue (0.4%; Invitrogen) in a ratio of 1:1 (10 μl: 10 μl) and quantified using Countess™ Cell Counting Chamber Slides (Invitrogen) and the Countess® Automated Cell Counter (Invitrogen) according to the manufacturer's instructions. The numbers of alive and dead cells were determined at each time point post-infection. All the primary macrophages were isolated from 6 individual fish and technical triplicates were utilized.

### RNA Extraction

RNA samples were obtained from head kidney primary macrophages inoculated with either PBS; live *A. salmonicida* (J223); formalin-killed *A. salmonicida* (FK J223); 100 ng ml^−1^ vitamin D_2_ or D_3_; 1,000 ng ml^−1^ vitamin D_2_ or D_3_, or 100 ng ml^−1^ vitamin D_2_ or D_3_ + live *A. salmonicida* (J223). The treatments that included vitamin D (D_2_ or D_3_) were pre-treated with the respective concentration 24 h before the challenge, meanwhile treatments without vitamin D were pre-treated only with the control vehicle 24 h before the challenge. Each sample was obtained 3 h post-inoculation.

Total RNA from Atlantic salmon primary macrophages was extracted using 1 ml of TRIzol Reagent (Invitrogen), and purified using the RNeasy® Mini Kit (QIAGEN) following the manufacturer's instructions (44). RNA samples were treated with 2 U of TURBO DNase (TURBO DNA-free™ Kit, Invitrogen) following the manufacturer's instructions to degrade any residual genomic DNA. Briefly, samples were incubated at 37°C for 30 min, 2.5 μl of DNase Inactivation Reagent was added, and samples incubated 5 min at room temperature. Then, samples were centrifuged at 10,000 x g for 1.5 min and the supernatant containing the RNA carefully transferred to a new tube. Purified RNA samples were quantified and evaluated for purity (A260/280 and A260/230 ratios) using a Nano-quant spectrophotometer (Genway, UK), and evaluated for integrity using 1% agarose gel electrophoresis (45). Column purified RNA samples had A260/280 ratios between 1.9 and 2.1 and A260/230 ratios between 1.9 and 2.2. A PCR test was conducted using the reference genes' primers [*60S ribosomal protein L32 (rpl32)* and β*-actin*] and the RNA as template to rule out the presence of DNA. All RNA samples did not show presence of DNA.

First-strand cDNA templates for qPCR were synthesized from 500 ng of DNaseI-treated, column-purified total RNA using SuperScript™ IV VILO™ Master Mix (Invitrogen) following the manufacturer's instructions. Each sample was incubated at 25°C for 10 min, at 50°C for 10 min, and at 85°C for 5 min.

### Gene Paralogue Discovery and qPCR

All qPCR reactions were performed in a 20 μl reaction, containing 1 × PowerUp SYBR Green Master Mix (Applied BioSystems, Foster City, CA, USA), 500 nM (final concentration) of both the forward and reverse primer and the indicated cDNA quantity. All samples were amplified and detected using the QuantStudio 3 Real Time PCR System (Applied BioSystems). The reaction mixtures were incubated for 2 min at 50°C, then 2 min at 95°C, followed by 40 cycles of 1 s at 95°C, 30 s at 60°C, and finally 15 s at 95°C, 1 min at 60°C, and 15 s at 95°C.

The primer sequences of *interleukin 1 beta* (*il-1b*), *interleukin 8 (il-8), tumor necrosis factor alpha (tnf-*α*), soluble toll-like receptor 5* (s*tlr5*), and *leukocyte-derived chemotaxin 2* (*lect-2*) are listed in Table 1. Gene paralogue discovery, qPCR primer design and initial quality testing were performed as described in Caballero-Solares et al. (49). Since the reagents, cycling conditions and samples were different in the current study, primer efficiencies (Table 1) were reassessed. Briefly, a 7-point 1:3 dilution series starting with cDNA representing 40 ng of input total RNA was generated, and efficiencies then calculated using the formula E = 10^(−1/slope)^ (50).

**Table 1:** Primers used in qPCR studies.

**Gene name (symbol) (GenBank acc. no.)**	**Nucleotide sequence (5^**′**^-3^**′**^)**	**^c^Efficiency (%)**	**Amplicon size (bp)**	**References**
interleukin 1 beta (*il-1b*) (AY617117)	F: GTATCCCATCACCCCATCAC	99.7	119	This study
	R: TTGAGCAGGTCCTTGTCCTT			
interleukin 8 (*il-8*) (BT046706)	F: GAAAGCAGACGAATTGGTAGAC	100.7	99	This study
	R: GCTGTTGCTCAGAGTTGCAAT			
tumor necrosis factor alpha (*tnf-α*) (AY929386)	F: GGATGGAATGGAGCATCAGC	106.4	141	(39)
	R: TGCACGGTGTTAGCGGTAAG			
leukocyte cell derived chemotaxin 2 (*lect-2*) (BT059281)	F: CAGATGGGGACAAGGACACT	101.1	150	(39)
	R: GCCTTCTTCGGGTCTGTGTA			
toll-like receptor 5 (soluble) (*stlr5*) (AY628755)	F: ATCGCCCTGCAGATTTTATG	94.1	103	(39)
	R: GAGCCCTCAGCGAGTTAAAG			
^a^β-actin (*actb*) (*tnf-α*) (BG933897)	F: CCAAAGCCAACAGGGAGAAG	104.4	91	(46)
	R: AGGGACAACACTGCCTGGAT			
^a^60S ribosomal protein *L32* (*rpl32*) (BT043656)	F: AGGCGGTTTAAGGGTCAGAT	100.7	119	(46)
	R: TCGAGCTCCTTGATGTTGTG			
^b^18S (*18S*)	F: GTCCGGGAAACCAAAGTC	91.0	Not provided	(47)
	R: TTGAGTCAAATTAAGCCGCA			
^b^elongation factor 1 alpha (*EF-1α*) (AF321836)	F: TGGCACTTTCACTGCTCAAG	96.3	197	(39)
	R: CAACAATAGCAGCGTCTCCA			
^b^hypoxanthine phosphoribosyl transferase 1 (*HPRT1*) (EG866745)	F: CCGCCTCAAGAGCTACTGTAAT	94.7	255	(48)
	R: GTCTGGAACCTCAAACCCTATG			

a *Normalizers used in experimental qPCR analyses*.

b *Candidate normalizer genes*.

c *Amplification efficiencies were calculated using a 7-point 1:3 dilution series starting with cDNA representing 40 ng of input total RNA. See methods for details*.

Transcripts levels of the genes of interest (*il-1b, il-8, tnf-*α, *stlr5*, and *lect-2*) were normalized to transcript levels of two endogenous control genes. Levels of five candidate normalizers (*60S ribosomal protein 32*; β*-actin, 18S, elongation factor 1 alpha*, and *hypoxanthine phosphoribosyl transferase 1*) were assessed in 50% of the samples (i.e., in 3 random samples per treatment) using cDNA representing 40 ng of input total RNA. Reference gene stability was then analyzed using both geNorm and BestKeeper (Supplementary Table 1). Both analyses identified β*-actin* (geNorm M = 0.592; BestKeeper value = 0.263) and *60S ribosomal protein L32* (geNorm M = 0.592; BestKeeper value = 0.364) and as the most stably expressed genes (Supplementary Table 1).

After normalizer testing was completed, transcript levels of the genes of interest were analyzed in the individual study samples, with normalization to both β*-actin* and *60S ribosomal protein L32*. In all cases, levels were assessed (in triplicate) in six individuals per treatment using cDNA representing 40 ng of input total RNA. On each gene a no RT control was included. Gene expression was determined using the comparative 2^−ΔΔCt^ method (51).

### Phagocytosis Assay

The phagocytosis assay was performed following the protocol used by Smith et al. (39) with modifications. Cells were incubated for 3 days and inoculated with vehicle control (2 μl of ethanol in 1 ml of culture media without antibiotics), 100; 1,000; or 10,000 ng ml^−1^ of either vitamin D_2_ or D_3_ for 24 h. After this time, cells were washed twice with PBS, and inoculated with 1 μm of Fluoresbrite YG microspheres at a ratio of ~1:30 macrophage:microsphere (Polysciences, Warrington, PA, USA) (39, 52). Twenty-four hours after microsphere addition, primary macrophages were washed with PBS and posteriorly cells treated with trypsin-EDTA (0.5%; Gibco) for 10 min. Then, cells were resuspended in 500 μl of FACS buffer (PBS + 1% FBS). Fluorescence was detected from 10,000 cells using a BD FACS Aria II flow cytometer and analyzed using BD FACS Diva v7.0 software (BD Biosciences, San Jose, CA, USA). The control pre-treated macrophages were used to compare with the FITC positive cells in vitamin D pre-treated cells. Percentages of FITC positive cells were determined for each condition. The experiments were conducted using macrophages isolated from 3 independent fish.

### Statistical Analysis

All data are shown as the mean ± standard error (SE). Assumptions of normality and homoscedasticity were tested for the detected variances. A Kruskal-Wallis non-parametric test was performed for *A. salmonicida* growth curve and gene expression results. Macrophages viability, gentamicin exclusion assay, and phagocytosis assay data were analyzed using a repeated measures two-way ANOVA test, followed by Sidak multiple comparisons *post hoc* test to identify significant differences of each treatment in different times or concentrations and between treatments in the same time point. Differences were considered significant at *P* < 0.05. All statistical analyses were performed using GraphPad Prism (GraphPad Software, La Jolla California USA, www.graphpad.com).

## Results

### Inhibitory Effects of Vitamin D_2_ and D_3_ on *A. salmonicida* Growth

**Figure 1:**
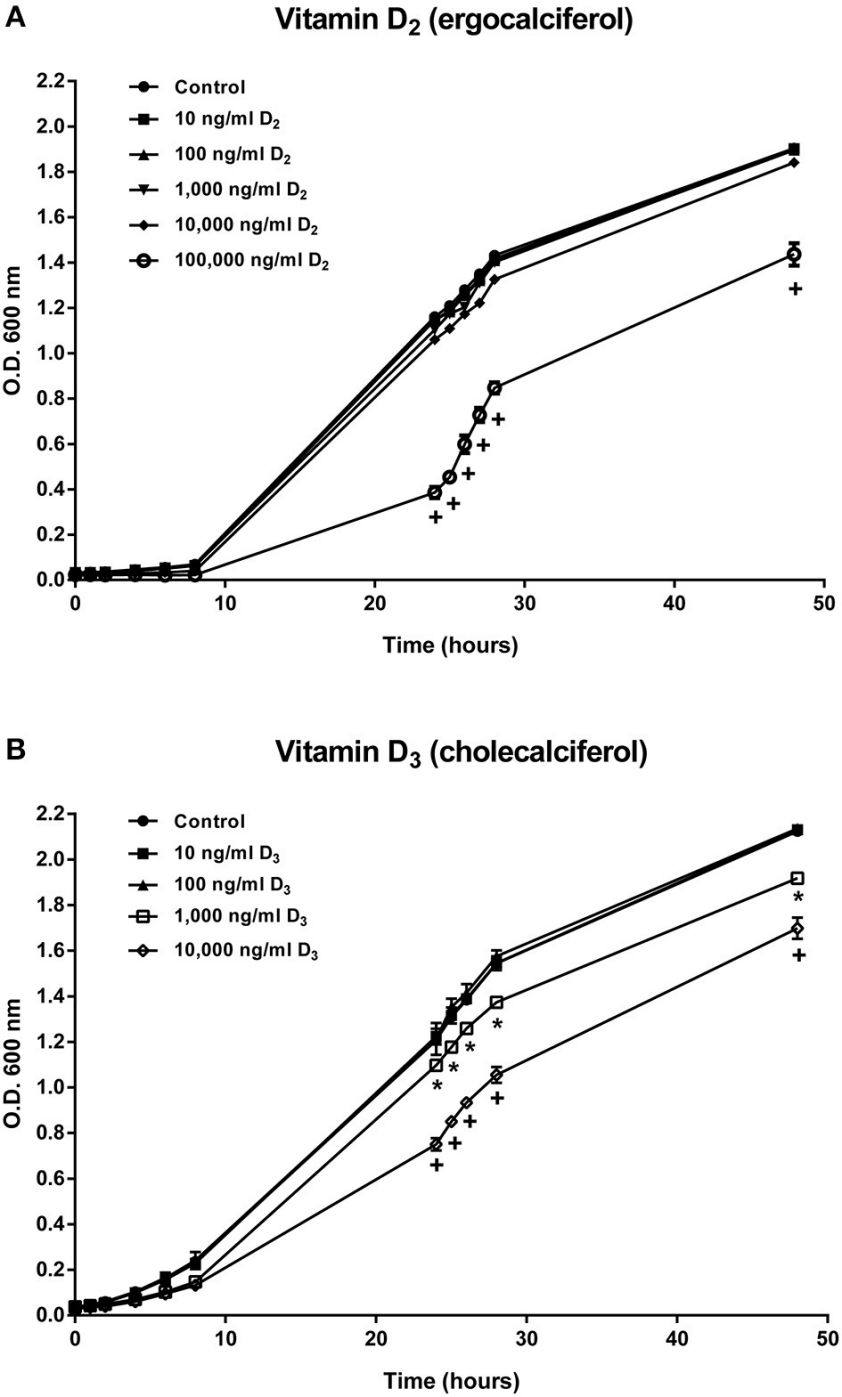
*Aeromonas salmonicida* subsp. *salmonicida* growth curve in TSB media supplemented with **(A)** 10, 100, 1,000, 10,000, and 100,000 ng ml^−1^ of vitamin D_2_ and **(B)** 10, 100, 1,000, and 10,000 ng ml^−1^of vitamin D_3_. Growth was determined by reading O.D. 600 nm at different time points until 48 h. Each value is the mean ± S.E.M (*n* = 3). Symbols (*, +) indicate differences between each group at different time points of measure, *p* < 0.05.

Growth of *A. salmonicida* at different concentrations of vitamin D_2_ and D_3_ was determined by O.D. 600 nm at different time points until 48 h. Bacteria growth was not affected in concentrations of 10, 100, 1,000, and 10,000 ng ml^−1^ of vitamin D_2_ (vitamin D_2_) (Figure 1A). In contrast, *A. salmonicida* growth was significantly reduced in the presence of 100,000 ng ml^−1^ of vitamin D_2_ (Figure 1A). *A. salmonicida* growth was not affected by 10 and 100 ng ml^−1^of vitamin D_3_ (vitamin D_3_) (Figure 1B). However, *A. salmonicida* growth was significantly affected by 1,000 and 10,000 ng ml^−1^ of the vitamin D_3_ (Figure 1B).

### Evaluation of the Toxicity of Vitamin D_2_ and D_3_ in Atlantic Salmon Primary Macrophages

**Figure 2:**
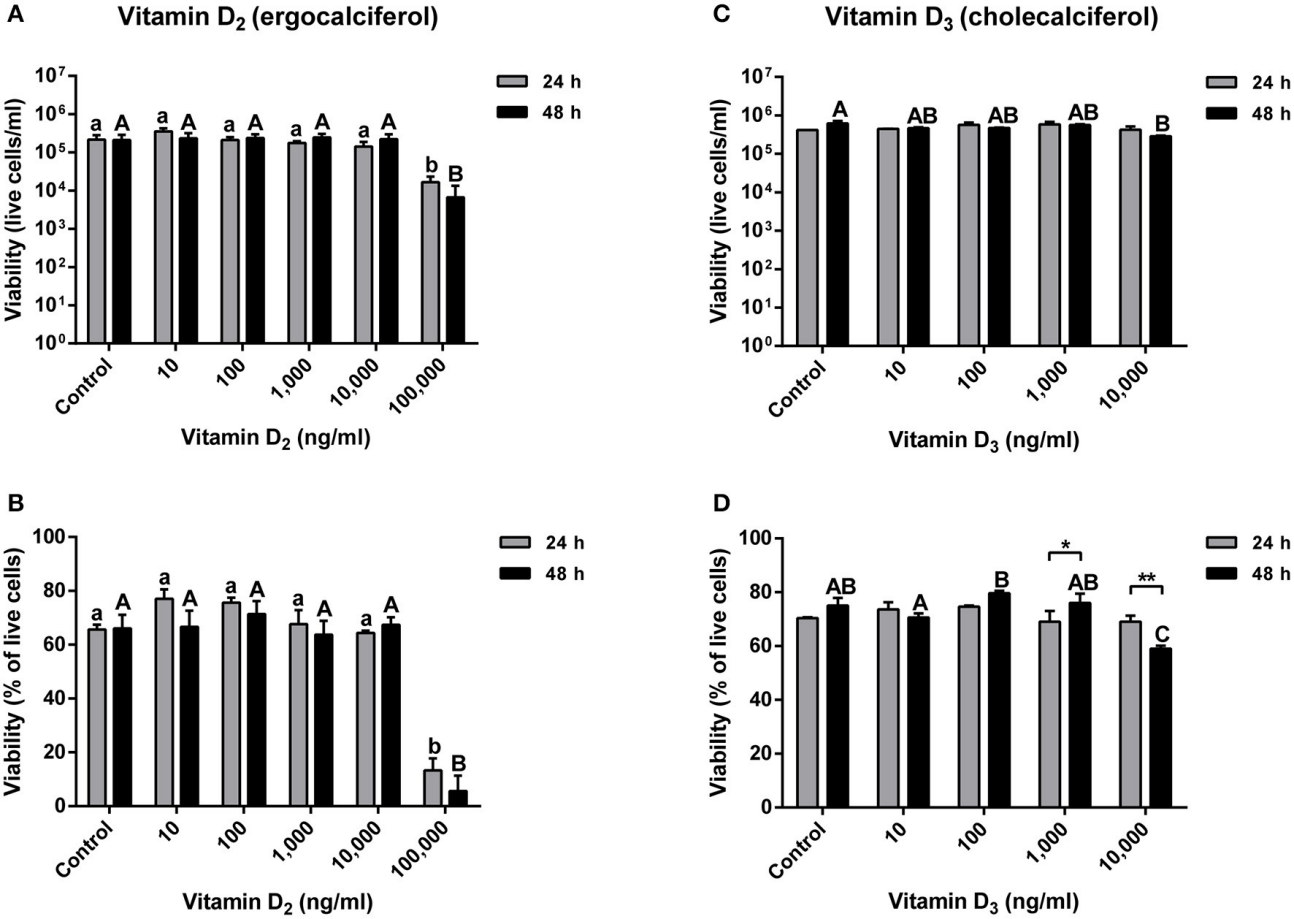
Atlantic salmon primary macrophages treated with vitamin D_2_ or D_3_. **(A)** Live cells and **(B)** percentage of viability of primary macrophages treated with 10, 100, 1,000, 10,000, and 100,000 of vitamin D_2_, were measured after 24 and 48 h of treatment. **(C)** Live cells and **(D)** percentage of viability of primary macrophages treated with 10, 100, 1,000, and 10,000 ng ml^−1^ of vitamin D_3_ were measured after 24 and 48 h of treatment. Each value represents the mean ± S.E.M (*n* = 6). Lower case letters (a, b) show differences between treatments after 24 h. Upper case letters (A,B,C) show differences between treatments after 48 h. Asterisks (*) represent significant differences between treatments (*p < 0.05, **p < 0.01).

Atlantic salmon primary macrophage viability was determined after 24 and 48 h of exposure to concentrations of 0, 10, 100, 1,000, 10,000, and 100,000 ng ml^−1^ of vitamin D_2_. Results obtained did not show significant differences in viability between the control group, compared with the cells treated with 10, 100, 1,000, and 10,000 ng ml^−1^ of vitamin D_2_. However, a significant difference was observed in cells inoculated with the media containing a concentration of 100,000 ng ml^−1^ of vitamin D_2_ after 24 h (1.67 × 10^4^ ± 6.67 × 10^3^) and 48 h (6.67 × 10^3^ ± 6.67 × 10^3^) (Figure 2A).

The percentage of viability did not show significant differences between the control group and the primary macrophages treated with 10, 100, 1,000, and 10,000 ng ml^−1^ of vitamin D_2_. Nevertheless, a highly significant decrease in macrophage viability was observed at a concentration of 100,000 ng ml^−1^ of vitamin D_2_ after 24 h (13.33 ± 4.48%) and 48 h (5.67 ± 5.67%) (Figure 2B).

The viability of Atlantic salmon primary macrophages was also determined at 24 and 48 h post-treatment with vitamin D_3_ in concentrations of 10, 100, 1,000, and 10,000 ng ml^−1^. The number of live cells per ml did not show significant differences in primary macrophages treated with 10, 100, and 1,000 ng ml^−1^ (Figure 2C). Moreover, no significant differences were observed in the viability of primary macrophages treated with 10,000 ng ml^−1^ of vitamin D_3_ after 24 h compared with the control (Figure 2C). Nevertheless, in cells exposed to 10,000 ng ml^−1^ of vitamin D_3_, a significant decrease in the viability was observed after 48 h of treatment (2.85 × 10^5^ ± 1.44 × 10^4^) (Figure 2C).

The percentage of viability in vitamin D_3_ treated cells did not show significant differences after 24 h of exposure to concentrations of 10, 100, 1,000, and 10,000 ng ml^−1^ compared with the control group (Figure 2D). Also, no significant differences were observed in Atlantic salmon macrophages treated during 48 h with vitamin D_3_ in a concentration of 10, 100, and 1,000 ng ml^−1^ compared with the control (Figure 2D). However, a significant decrease was observed in cells incubated with a concentration of 10,000 ng ml^−1^ of vitamin D_3_ compared with the control and all the lower doses of vitamin D_3_ at the 48 h time point (59.00 ± 1.15%) (Figure 2D). Moreover, in the group incubated at a concentration of 1,000 ng ml^−1^ of vitamin D_3_, a significant higher percentage of viability was observed in cells treated for 48 h compared with the 24 h group (Figure 2D). Also, in the primary macrophages treated with 10,000 ng ml^−1^ of vitamin D_3_, a significant difference was observed at different times, showing a decrease in the viability of cells treated for 48 h with vitamin D_3_ compared with the cells incubated for only 24 h (Figure 2D).

### Gentamicin Exclusion Assay in Vitamin D_2_ and D_3_ Pre-treated Cells Infected With *A. salmonicida*

The effects of vitamins D_2_ and D_3_ on the growth of *A. salmonicida* (Figure 1) and the effects on the viability of primary macrophages (Figure 2) were used to determine the concentration to be utilized in the gentamicin exclusion assays. Based on these results, the primary macrophages were pre-treated with 100 ng ml^−1^ (vitamin D_2_ or D_3_) for the gentamicin exclusion assay.

**Figure 3:**
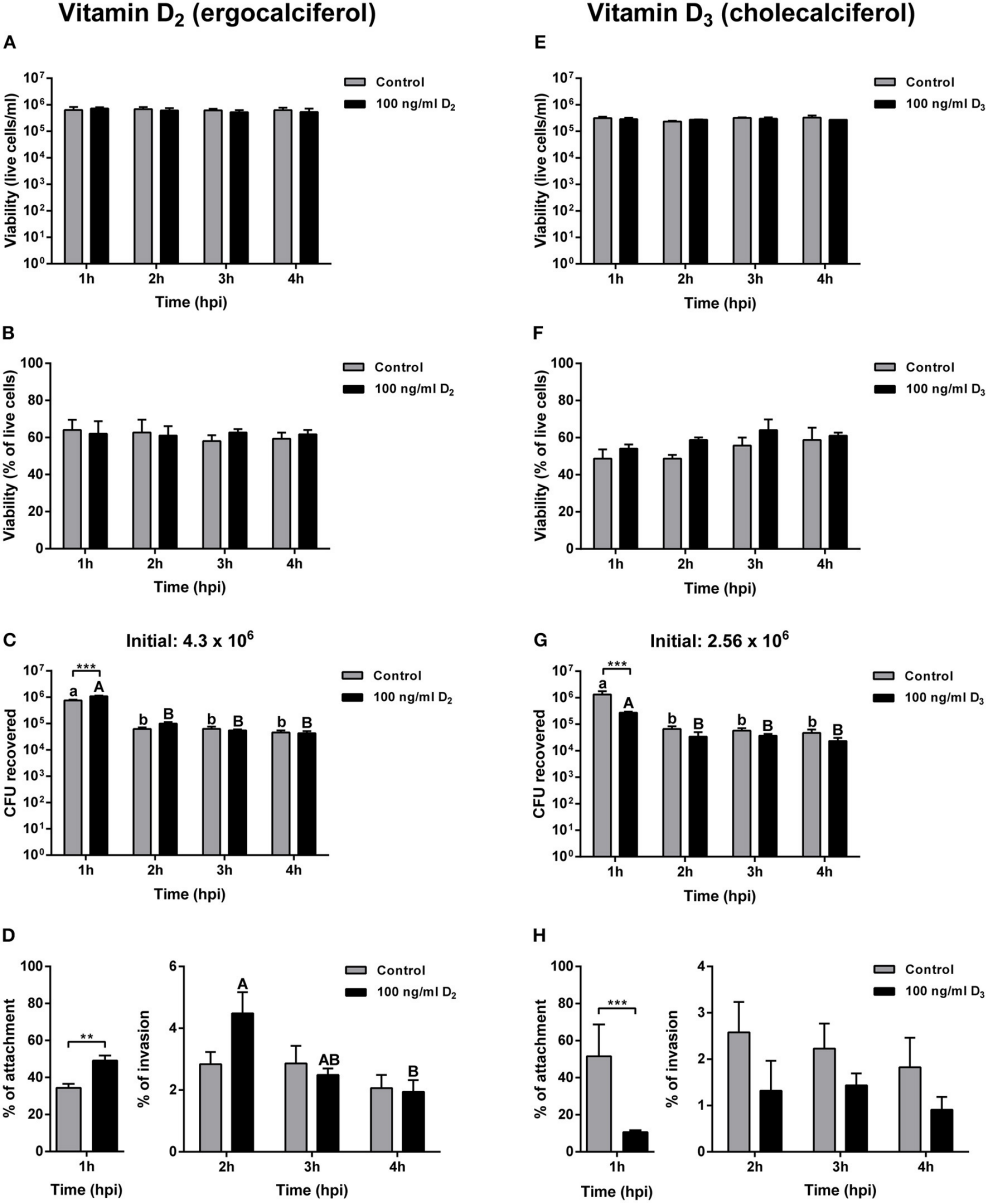
Gentamicin exclusion assay in Atlantic salmon primary macrophages pre-treated for 24 h with either control or 100 ng ml^−1^ of vitamin D_2_ or vitamin D_3_, and then infected with *Aeromonas salmonicida* subsp. *salmonicida*. Live cells of primary macrophages pre-treated with vitamin D_2_ **(A)**, or vitamin D_3_ **(E)**; percentage of viability of primary macrophages pre-treated with vitamin D_2_ **(B)**, or vitamin D_3_ **(F)**, Colony forming unit (CFU) of *A. salmonicida* in Atlantic salmon primary macrophages pre-treated with vitamin D_2_ **(C)**, or vitamin D_3_ **(G)**; and percentage of attachment and invasion of *A. salmonicida* in Atlantic salmon primary macrophages pre-treated with vitamin D_2_ **(D)**, or vitamin D_3_ **(H)**, were measured 1, 2, 3, and 4 h post-infection. Initial *A. salmonicida* inoculum calculated in TSA Congo red plates are shown above the CFU figures. Each value represents the mean ± S.E.M (*n* = 6). Asterisks (*) represent significant differences between treatments on each time-point (**p < 0.01, ***p < 0.001). Lower case letters (a, b) show differences in the control at different time points post-infection. Upper case letters (A, B) show differences in vitamin D_2_ or D_3_ pre-treated cells in different time points post-infection.

Cells pre-treated for 24 h with 100 ng ml^−1^ of vitamin D_2_ and then infected with *A. salmonicida*, did not show significant differences in cell numbers at 1, 2, 3, and 4 h post-infection compared with the control group (Figure 3A). Also, no significant differences were observed in the viability of primary macrophages pre-treated and then infected with *A. salmonicida* at 1, 2, 3, and 4 h post-infection compared with the control group (Figure 3B).

The primary macrophages were infected with a total of 4.3 × 10^6^ CFU per ml at a MOI of 1. The percentage of *A. salmonicida* attached was significantly higher in primary macrophages pre-treated with vitamin D_2_ (49.09 ± 2.76%) compared with the control group (34.39 ± 2.12%) (Figure 3D). At invasion time-points, after 2 h of infection no significant differences were observed in cells pre-treated with vitamin D_2_ compared with the control group at the same time point (Figure 3D). Moreover, no significant differences were observed in the control group and the vitamin D_2_ pre-treated primary macrophages after 3 h of infection with *A. salmonicida* (Figure 3D). No significant differences were also found between the control and the vitamin D_2_ treatment 4 h post-infection. However, a significant decrease in bacterial invasion was observed between 2 and 4 h in the primary macrophages pre-treated with vitamin D_2_ (Figure 3D).

A similar response was obtained in cells pre-treated for 24 h with vitamin D_3_ and then infected with *A. salmonicida*. For instance, no significant differences were found in the percentage of viability between the control group after 1, 2, 3, and 4 h of infection and the vitamin D_3_ pre-treated macrophages at 1, 2, 3, and 4 h (Figure 3F).

Atlantic salmon primary macrophages pre-treated with vitamin D_3_ were infected with a total of 2.56 × 10^6^ bacterial cells per ml^−1^ (Figure 3G). The percentage of attachment (1 h post-infection) was significantly lower in the primary macrophages pre-treated for 24 h with vitamin D_3_ (10.61 ± 0.97%) compared with the control group (51.56 ± 17.12%) (Figure 3H). In contrast, even when a tendency of lower invasion is observed, no significant differences were observed between the control group after 2, 3, and 4 h of infection compared with the fish cells pre-treated with vitamin D_3_ (Figure 3H).

### Atlantic Salmon Primary Macrophages Gene Expression

Transcript levels of innate immune response-related genes were evaluated by qPCR in Atlantic salmon primary macrophages after 3 h of the aforementioned treatments.

**Figure 4:**
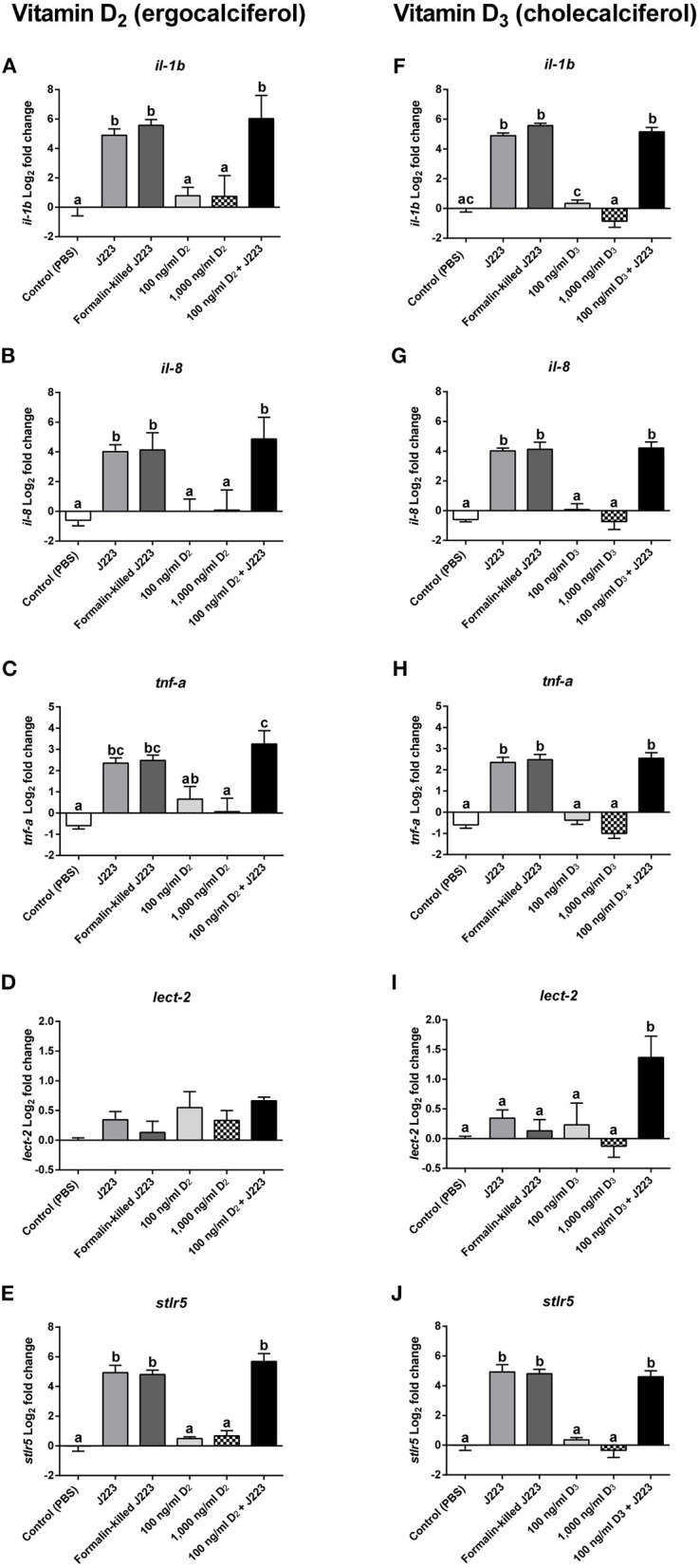
Gene expression of **(A,F)** *Interleukin 1b* (*il-1b*), **(B,G)** *Interleukin 8* (*il-8*), **(C,H)** *Tumor necrosis factor alpha* (*tnf-*α), **(D,I)** *Leukocyte-derived chemotaxin 2* (*lect-2*), and **(E,J)** *soluble toll-like receptor 5* (s*tlr5*) in Atlantic salmon primary macrophages isolated from head kidney pre-treated 24 h with either the control, vitamin D_2_ (100 and 1,000 ng ml^−1^) or vitamin D_3_ (100 and 1,000 ng ml^−1^), and then inoculated with PBS (control) or infected with live (J223) or exposed to formalin-killed *A. salmonicida* for 3 h. Relative expression was calculated using the 2^(−ΔΔCt)^ method and Log_2_ converted using β*-actin* and *60S ribosomal protein L32* (*rpl32*) as internal reference genes. Each value is the mean ± S.E.M (*n* = 6). Different letters represent significant differences between treatments, *p* < 0.05.

In the experiments conducted for both vitamin D_2_ and D_3_, a significant increase in the transcript expression of *il-1b* (Figures 4A,F), *il-8* (Figures 4B,G), *tnf-*α (Figures 4C,H), and s*tlr5* (Figures 4E,J) was observed in cells inoculated with live *A. salmonicida*, formalin-killed *A. salmonicida*, and in cells pre-treated with either vitamin D_2_ or D_3_ and then infected, compared to the PBS inoculated primary macrophages. In contrast, no differences in the expression of *il-1b, il-8, tnf-*α, and s*tlr5* were observed in Atlantic salmon cells inoculated only with 100 ng ml^−1^ or 1,000 ng ml^−1^ of each vitamin D form compared with the control (Figures 4A–C,E–H,J).

A different pattern was observed in the expression of *lect-2* transcript between both treatments. For instance, in cells pre-treated with vitamin D_2_, no significant differences were observed in the expression of *lect-2* in any of the treatments compared with the PBS inoculated primary macrophages (Figure 4D). In contrast, *lect-2* was significantly up-regulated compared with the control in primary macrophages pre-treated with vitamin D_3_ and then challenged with *A. salmonicida* (Figure 4I). The primary macrophages treated with live *A. salmonicida*, formalin-killed *A. salmonicida*, 100 ng ml^−1^ of vitamin D_3_, and 1,000 ng ml^−1^ of vitamin D_3_ did not show significant differences in the expression of *lect-2* compared with the control (Figure 4I).

### Phagocytosis Assay

Atlantic salmon primary macrophages did not show significant differences in phagocytosis after 24 h of treatment with 100 ng ml^−1^ of vitamin D_2_ (4.80 ± 1.62%) and vitamin D_3_ (4.93 ± 1.56%), 1,000 ng ml^−1^ of vitamin D_2_ (3.33 ± 0.67%) and vitamin D_3_ (2.67 ± 0.41%), and 10,000 ng ml^−1^ of vitamin D_2_ (0.97 ± 0.20%) and vitamin D_3_ (0.87 ± 0.09%) compared with the control cells (4.80 ± 1.08%) (Supplementary Figure 1).

## Discussion

Vitamin D is involved in important processes including mineral metabolism, cell growth, and cardiovascular physiology, among others (1–6). Moreover, it has been observed that vitamin D can stimulate the antibacterial immune response in mammals (7, 8). However, these effects and mechanisms have not been explored in fish cells. The two major sources of vitamin D in natural environments are vitamin D_2_ and D_3_, being obtained in fish after the ingestion of phytoplankton and zooplankton, respectively (9, 10). Different from terrestrial vertebrates that rely on the conversion of 7-dehydrocholesterol to vitamin D by using solar ultraviolet light (wavelength 380–500 nm) through the skin, fish seems to have alternative mechanims to obtaining vitamin D (4). For instance, it has been hypothesized that bony scales that are part of the fish skin are able to focus and convert the photons obtained from the blue light to breakdown the 7-dehydrocholesterol into vitamin D (53). This does not mean that fish lack of the mechanisms for photosynthesis of vitamin D through the skin. For instance, has been reported that rainbow trout (*Oncorhynchus mykiss*) and Mozambique tilapia (*Tilapia mossambicus*) have the mechanisms to convert 7-dehydrocholesterol into vitamin D, however, authors concluded that in natural environments photosynthesis of vitamin D does not play a significant role (4, 9, 54).

Initially it was thought that both vitamin D forms had the same impact on physiology (55), however, several studies have shown that vitamin D_3_ is much more potent compared with vitamin D_2_ (55, 56). This evidence suggests a differential modulation on the physiology of fish (i.e., innate immune system) in the presence of the specific vitamin D forms. Also, the effect that vitamin D forms can have on the growth of *A. salmonicida* has not yet been described.

To evaluate if *A. salmonicida* is able to grow in the presence of vitamin D_2_ and D_3_, a growth curve experiment was conducted in the presence of different concentrations of vitamin D_2_ and vitamin D_3_ for 48 h. Our results showed that only high concentrations of vitamin D_2_ and D_3_ reduced the growth of *A. salmonicida* after 48 h (Figures 1A,B). Normally, *A. salmonicida* is able to reach the stationary growth phase in ~36 h (24, 57, 58). We observed a similar pattern of growth previously observed in *A. salmonicida* J223 strain (24) in culture media containing low concentrations of vitamin D_3_ (100 and 1,000 ng ml^−1^). Nevertheless, *A. salmonicida* seemed to tolerate higher concentrations of vitamin D_2_ since only the highest concentration (100,000 ng ml^−1^) reduced its growth rate. A previous study showed that high doses of vitamin C (128, 512, and 2048 mg/ml) can inhibit the growth of *Helicobacter pylori*, a risk factor for gastric carcinoma in mammals, during *in vitro* and *in vivo* experiments (59). Additionally, it has been reported that high concentrations of vitamin C (90 μM) can also inhibit the growth of *S. aureus* in *in vitro* conditions (60). High concentrations of vitamin D_2_ and D_3_ decreased the growth rate of *S. aureus* strain A1 after 24 h (14, 61). However, no significant differences were observed in the growth rate of *S. aureus* subsp. *aureus* (ATCC 27543) in the presence of different concentrations of vitamin D_3_ after 48 h (8, 62). These results indicate that in the bacterial strains studied, vitamin D can inhibit growth when it is utilized in concentrations over 1,000 ng ml^−1^, affecting both Gram negative and Gram positive bacteria.

The primary Atlantic salmon macrophage viability decreased after 24 and 48 h of exposure to 100,000 ng ml^−1^ of vitamin D_2_, and a lower viability compared with the control was observed after 48 h with 10,000 ng ml^−1^ of vitamin D_3_ (Figure 2). Similar to our results, it has been observed that low concentrations of vitamin D_3_ (1, 10, and 50 nM) did not affect the viability of bovine mammary epithelial cells at 24 h (8). However, it has been reported that a decrease in the viability of bovine mammary epithelial cells in the presence of high concentrations of vitamin D_2_ (6,000, 8,000, 10,000, 12,000, and 14,000 ng ml^−1^) and D_3_ (8,000, 10,000, 12,000, and 14,000 ng ml^−1^) occurred after 24 h of exposure, suggesting that vitamin D can induce cell cycle arrest, apoptosis, or both (14, 63).

In Atlantic salmon, the estimated concentration of vitamin D in flesh varies between 2.9 μg to 18.5 μg per 100 g^−1^ (64). In our studies, the vitamin D concentrations utilized were similar to other studies conducted in mammalian cells used as a reference (8, 13, 14). The values obtained in the *A. salmonicida* growth curve and Atlantic salmon primary macrophages exposed to different concentrations of vitamin D_2_ and D_3_ (Figures 1, 2), were utilized to determine the final non-toxic vitamin concentration (100 ng ml^−1^) for further infection assays. The number of live cells and percentage of viability of Atlantic salmon primary macrophages after 1, 2, 3, and 4 h of infection did not show significant differences between the control and the vitamin D_2_ or D_3_ pre-treated cells (Figures 2A,B,E,F). Our findings agree with previous infection assays using *A. salmonicida* J223 in Atlantic cod primary macrophages, where no significant differences were observed in macrophage viability after 6 h of infection (42). This indicated that Atlantic salmon and Atlantic cod primary macrophages were not killed during this period by *A. salmonicida* J223. We previously suggested (42) that *A. salmonicida* controls the macrophages machinery to prevent cell apoptosis. As mentioned previously, vitamin D_3_ in high concentration (8,000, 10,000, 12,000, and 14,000 ng ml^−1^) could induce apoptosis (14, 63), perhaps in opposition to this suggested prevention of apoptosis produced by *A. salmonicida* (42). These results agree with the lower attachment and infection rates of *A. salmonicida* in cells pre-treated with D_3_, where vitamin D_3_ might interfere with the infection.

One of the most interesting findings of our results is related to the bacterial attachment and invasion. For instance, a significant increase in *A. salmonicida* infection was observed at 1 h post-infection in primary macrophages pre-treated with vitamin D_2_ compared with the control (Figures 3C,D). In contrast, a significant decrease in *A. salmonicida* attachment at 1 h was observed in cells pre-treated with vitamin D_3_ compared with the control (Figures 3G,H). Previous studies indicated that vitamin D_3_ has a stronger activity compared to vitamin D_2_ in terrestrial mammals (55, 56). This agrees with our results where pre-treatment of primary macrophages with vitamin D_3_ decreased *A. salmonicida* infection; while vitamin D_2_ in contrast, seems to increase it infection. Our findings agree with the beneficial utilization of vitamin D_3_ in aquafeeds (4, 65, 66) and with vitamin D_3_ potentially having a broad positive effect in all vertebrates, including fish.

To complement our results described above, we explored part of the immune mechanism behind the beneficial effects of vitamin D by profiling the expression of specific innate immune genes using qPCR. An up-regulation of *il-1b, il-8, tnf-*α, and s*tlr5* occurred in primary macrophages inoculated with either live or formalin-killed *A. salmonicida* (Figures 4A–C,E–H,J). These results were expected, since cytokines and chemokines, such as *il-1b, il-8, tnf-*α, and the pattern recognition receptor (PRR) s*tlr5*, play essential roles controlling both acute and chronic inflammation in fish tissues mediated by macrophages (39). In Atlantic salmon, the evidence shows that this canonical macrophage innate immune response can be triggered rapidly by either a bacterial pathogen-associated molecular pattern (PAMP), such as lipopolysaccharide (LPS), or pathogens like *Yersinia ruckeri, Aeromonas salmonicida, Pseudomonas aeruginosa*, and *Flavobacterium psychrophilum*, among others (39, 67–70).

Some viruses, bacteria, and parasites can modify the expression of genes related with the host immune response as part of a mechanism of evading its defense mechanisms (71). In humans, three important infectious diseases (e.g., HIV, tuberculosis, and malaria) have developed highly effective mechanisms to subvert the immune response (71), making it difficult to control these diseases and develop effective vaccines. Further examples of this are *Yersinia pseudotuberculosis* and *Y. enterocolitica* which are able to control human macrophage immune response and induce apoptosis after the translocation of effector molecules through the type III secretion system (72–74).

In fish head kidney, a modulation of the expression of *il-1b* and the *major histocompatibility complex class 1* (*mhc-I*) has been observed in Atlantic salmon after being infested by the sea louse *Lepeophtheirus salmonis* (75). Moreover, Lewis et al. (76) showed in an Atlantic salmon head kidney (SHK-1) cell line that *L. salmonis* produces substances that modify the expression of genes encoding inflammatory mediators. Here, our results obtained during the infection with live *A. salmonicida* in Atlantic salmon primary macrophages showed no significant differences in the expression of *leukocyte-derived chemotaxin 2* (*lect-2)* compared with the control (Figures 4D,I). When the fish cells were pre-treated with vitamin D_2_ and then infected with live bacteria, the gene expression also did not increase, suggesting that *lect-2* is not involved in the first line of defense against *A. salmonicida* in Atlantic salmon. However, the expression of *lect-2* in Atlantic salmon macrophages pre-treated with vitamin D_3_ and challenged with live *A. salmonicida* was significantly up-regulated compared to the control (Figure 4I). *lect-2* is a chemotactic factor involved in the recruitment of neutrophils to the site of infection (39, 77). In the study conducted by Smith et al. (39), an up-regulation of *lect-2* was observed only in treatments with LPS, confirming that its role in the presence of external pathogenic agents is active in Atlantic salmon. Comparing the expression of *lect-2* in primary macrophages treated with live *A. salmonicida* and the samples pre-treated with vitamin D_3_ and then inoculated with *A. salmonicida*, our results suggest that *A. salmonicida* prevent the transcriptional response of *lect-2*, perhaps to prevent neutrophil recruitment during infection. Our results suggest that pre-treatments with vitamin D_3_ can counteract the effect of *A. salmonicida* on this particular gene, and up-regulate the expression of *lect-2* during *A. salmonicida* infection (Figure 4I).

To determine if vitamin D_2_ and D_3_ can also cause an effect on the phagocytosis of Atlantic salmon primary macrophages, the phagocytic activity was tested using fluorescent latex beads (Supplementary Figure 1). Phagocytosis is used by organisms to eliminate external agents such as bacteria in a highly efficient way (42, 52, 78, 79). The effect of vitamins on macrophages' phagocytic activity has been previously tested in Atlantic salmon, however, no significant variations were observed after treatments with vitamin C or vitamin E (80, 81). We found similar results in Atlantic salmon primary macrophages after 24 h pre-treatments with either vitamin D_2_ or D_3_, suggesting that, independent of the vitamin D used, the phagocytic activity of Atlantic salmon macrophages is not modulated by its action.

## Conclusion

In this study we evaluated the effects of vitamin D_2_ and D_3_ on *A. salmonicida* growth, Atlantic salmon primary macrophage viability, and the fish cells' immune response. We determined that only high concentrations of vitamin D_2_ (100,000 ng ml^−1^) and vitamin D_3_ (1,000 and 10,000 ng ml^−1^) decreased the growth rate of *A. salmonicida*. Moreover, we determined that 100,000 ng ml^−1^of vitamin D_2_ and 10,000 ng ml^−1^ of vitamin D_3_ decreased the viability of Atlantic salmon primary macrophages after 24 and 48 h. These results suggest that high doses of D_2_ and D_3_ are toxic for the bacterial and the eukaryotic cells.

Pre-treatment of primary macrophages with 100 ng ml^−1^of either vitamin D_2_ or D_3_ did not have effects on cell viability. Nevertheless, one of the remarkable findings of our study was that pre-treatment with vitamin D_3_ reduced *A. salmonicida* attachment, meanwhile, pre-treatment with vitamin D_2_ increased attachment, and as a consequence also increased bacterial invasion.

Gene expression of *il-1b, il-8, tnf-*α, and s*tlr5* was up-regulated during *A. salmonicida* infection, agreeing with a canonical innate immune response. However, our results suggested that *A. salmonicida* was able to suppress the expression of *lect-2*, a gene involved in neutrophil recruitment, key in the fight against pathogen clearance. After the addition of vitamin D_2_, no variation in the transcriptional expression of this gene was observed. However, cells pre-treated with vitamin D_3_ and then inoculated with live *A. salmonicida*, showed an up-regulation of *lect-2*, suggesting that vitamin D_3_ can be useful to counteract the suppression triggered by the pathogen.

Altogether, our results show that vitamin D_3_ seems to be a good candidate to be used as an immunostimulant in Atlantic salmon against *A. salmonicida* infection. The mechanisms on how vitamin D_3_ modulates the *S. salar* macrophage immunity and its relation to specific receptors, like the vitamin D receptor, deserve future attention. In contrast, vitamin D_2_ did not appear to have an effect on the modulation of the immune system of Atlantic salmon, suggesting that vitamin D_2_ may not play an important role in the fish innate antibacterial immune response.

## Data Availability Statement

All datasets generated for this study are included in the article/Supplementary Material.

## Ethics Statement

The animal study was reviewed and approved by the Memorial University Institutional Animal Care Committee.

## Author Contributions

MS-D and JS: conception and design of research, prepared figures, and drafted the manuscript. MS-D: macrophage isolation. JH: primer design. MS-D, KV, and SI: performed the experiments. MS-D, MR, and JS: interpreted the results of the experiments. MS-D, KV, SI, JH, MR, and JS: edited and revised the manuscript and approved the final version of the manuscript. MR and JS: funding support.

### Conflict of Interest

The authors declare that the research was conducted in the absence of any commercial or financial relationships that could be construed as a potential conflict of interest.

## References

[1] Zittermann A. Vitamin D in preventive medicine: are we ignoring the evidence? Br J Nutr. (2003) 89:552–72. 10.1079/BJN2003837 12720576

[2] Grant W. Epidemiology of disease risks in relation to vitamin D insufficiency. Prog Biophys Mol Bio. (2006) 92:65–79. 10.1016/j.pbiomolbio.2006.02.013 16546242

[3] Lips P. Vitamin D physiology. Prog Biophys Mol Bio. (2006) 92:4–8. 10.1016/j.pbiomolbio.2006.02.016 16563471

[4] Lock EJ Waagbø R Wendelaar-Bonga S Flick G The significance of vitamin D for fish: a review. Aquac Nutr. (2010) 16:100–16. 10.1111/j.1365-2095.2009.00722.x

[5] Borges M Martini L Rogero M. Current perspectives on vitamin D, immune system, and chronic diseases. Nutrition. (2011) 27:399–404. 10.1016/j.nut.2010.07.022 20971616

[6] Wang L Xu H Wang Y Wang C Li J Zhao Z. Effects of the supplementation of vitamin D_3_ on the growth and vitamin D metabolites in juvenile Siberian sturgeon (*Acipenser baerii*). Fish Physiol Biochem. (2017) 43:901–9. 10.1007/s10695-017-0344-5 28116596

[7] Miller J Gallo R. Vitamin D and innate immunity. Dermatol Ther. (2010) 23:13–22. 10.1111/j.1529-8019.2009.01287.x 20136905

[8] Téllez-Pérez A Alva-Murillo N Ochoa-Zarzosa A López-Meza J. Cholecalciferol (vitamin D) differentially regulates antimicrobial peptide expression in bovine mammary epithelial cells: Implications during *Staphylococcus aureus* internalization. Vet Microbiol. (2012) 160:91–8. 10.1016/j.vetmic.2012.05.007 22655972

[9] Rao DS Raghuramulu N Food Chain as Origin of Vitamin D in Fish. Comp Biochem Physiol A Mol Integr Physiol. (1996) 114:15–9. 10.1016/0300-9629(95)02024-1

[10] Darias M Mazurais D Koumoundouros G Cahu C Zambonino-Infante J Overview of vitamin D and C requirements in fish and their influence on the skeletal system. Aquaculture. (2011) 315:49–60. 10.1016/j.aquaculture.2010.12.030

[11] Mora JR Iwata M von Andrian U. Vitamin effects on the immune system: vitamins A and D take centre stage. Nat Rev Immunol. (2008) 8:685–98. 10.1038/nri2378 19172691 PMC2906676

[12] Prentice A Goldberg G Schoenmakers I. Vitamin D across the lifecycle: physiology and biomarkers. Am J Clin Nutr. (2008) 88:500S−6. 10.1093/ajcn/88.2.500S 18689390

[13] Alva-Murillo N Téllez-Pérez A Medina-Estrada I Álvarez-Aguilar C Ochoa-Zarzosa A López-Meza J. Modulation of the inflammatory response of bovine mammary epithelial cells by cholecalciferol (vitamin D) during *Staphylococcus aureus* internalization. Microb Pathog. (2014) 77:24–30. 10.1016/j.micpath.2014.10.006 25457796

[14] Yue Y Hymøller L Jensen S Lauridsen C Purup S. Effects of vitamin D and its metabolites on cell viability and *Staphylococcus aureus* invasion into bovine mammary epithelial cells. Vet Microbiol. (2017) 203:245–51. 10.1016/j.vetmic.2017.03.008 28619151

[15] Sadarangani S Whitaker J Poland G “Let There Be Light”: The role of Vitamin D in the immune response to vaccines. Expert Rev Vaccines. (2015) 14:1427–40. 10.1586/14760584.2015.1082426 26325349 PMC4913549

[16] Asche F Roll K Sandvold H Sørvig A, Zhang D. Salmon aquaculture: Larger companies and increased production. Aquacult Econ Manage. (2013) 17:322–39. 10.1080/13657305.2013.812156

[17] Liu Y Rosten T Henriksen K Hognes E Summerfelt S Vinci B Comparative economic performance and carbon footprint of two farming models for producing Atlantic salmon (*Salmo salar*): Land-based closed containment system in freshwater and open net pen in seawater. Aquacult Eng. (2016) 71:1–12. 10.1016/j.aquaeng.2016.01.001

[18] FAO (2018). The State of World Fisheries and Aquaculture (SOFIA) - Meeting the Sustainable Development Goals, Food and Agriculture Organization, Rome, Italy.

[19] Fryer J.L. Sanders JE. Bacterial kidney disease of salmonid fish. Ann. Rev. Microbiol. (1981) 35, 273–298. 10.1146/annurev.mi.35.100181.001421 6794423

[20] Cvitanich JD Gárate O Smith CE The isolation of a Rickettsia-like organism causing disease and mortality in Chilean salmonids and its confirmation by Koch's postulate. J Fish Dis. (1991) 14:121–45. 10.1111/j.1365-2761.1991.tb00584.x

[21] Toranzo AE Magariños B Romalde JL A review of the main bacterial fish diseases in mariculture systems. Aquaculture. (2005) 246:37–61. 10.1016/j.aquaculture.2005.01.002

[22] Higuera G Bastías R Tsertsvadze G Romero J Espejo R Recently discovered *Vibrio anguillarum* phages can protect against experimentally induced vibriosis in Atlantic salmon, *Salmo salar*. Aquaculture. (2013) 392–395:128–33. 10.1016/j.aquaculture.2013.02.013

[23] Maisey K Montero R Christodoulides M. Vaccines for Piscirickettsiosis (salmonid rickettsial septicaemia, SRS): The Chile perspective. Expert Rev Vaccines. (2016) 16:215–28. 10.1080/14760584.2017.1244483 27690686

[24] Valderrama K Saravia M Santander J. Phenotype of *Aeromonas salmonicida* sp. *salmonicida* cyclic adenosine 3',5'-monophosphate receptor protein (Crp) mutants and its virulence in rainbow trout (*Oncorhynchus mykiss*). J Fish Dis. (2017) 40:1849–56. 10.1111/jfd.12658 28548689

[25] Kaatari S Tripp R Cellular mechanisms of glucocorticoid immunosuppression in salmon. J Fish Biol. (1987) 31:129–32. 10.1111/j.1095-8649.1987.tb05304.x

[26] Robertson L Thomas P Arnold C Trant J Plasma cortisol and secondary stress responses of red drum to handling, transport, rearing density, and a disease outbreak. Prog Fish Cult. (1987) 49:1–12.

[27] Siwicki AK Anderson DP Rumsey GL. Dietary intake of immunostimulants by rainbow trout affects non-specific immunity and protection against furunculosis. Vet Immunol Immunopathol. (1994) 41:125–39. 10.1016/0165-2427(94)90062-0 8066989

[28] Murray AL Pascho RJ Alcorn SW Fairgrieve WT Shearer KD Roley D Effects of various feed supplements containing fish protein hydrolysate or fish processing by-products on the innate immune functions of juvenile coho salmon (*Oncorhynchus kisutch*). Aquaculture. (2003) 220:643–53. 10.1016/S0044-8486(02)00426-X

[29] Dawood MA Koshio S Esteban MA Beneficial roles of feed additives as immunostimulants in aquaculture: a review. Rev Aquacult. (2017) 10:950–74. 10.1111/raq.12209

[30] Sakai M Current research status of fish immunostimulants. Aquaculture. (1999) 172:63–92. 10.1016/S0044-8486(98)00436-0

[31] Oliva-Teles A. Nutrition and health of aquaculture fish. J Fish Dis. (2012) 35:83–108. 10.1111/j.1365-2761.2011.01333.x 22233511

[32] Cook MT Hayball PJ Hutchinson W Nowak BF Hayball JD. Administration of a commercial immunostimulant preparation, EcoActiva^TM^ as a feed supplement enhances macrophage respiratory burst and the growth rate of snapper (*Pagrus auratus*, Sparidae (Bloch and Schneider)) in winter. Fish Shellfish Immun. (2003) 14:333–45. 10.1006/fsim.2002.0441 12657536

[33] Bridle A Carter C Morrison R Nowak B. The effect of β-glucan administration on macrophage respiratory burst activity and Atlantic salmon, *Salmo salar* L., challenged with amoebic gill disease – evidence of inherent resistance. J Fish Dis. (2005) 28:347–56. 10.1111/j.1365-2761.2005.00636.x 15960658

[34] Song SK Beck BR Kim D Park J Kim J Kim HD. Prebiotics as immunostimulants in aquaculture: a review. Fish Shellfish Immunol. (2014) 40:40–8. 10.1016/j.fsi.2014.06.016 24973515

[35] Secombes CJ Fletcher TC The role of phagocytes in the protective mechanisms of fish. Annu Rev Fish Dis. (1992) 2:53–71. 10.1016/0959-8030(92)90056-4

[36] Esteban MA Cuesta A Chaves-Pozo E Meseguer J. Phagocytosis in Teleosts. Impl N Cells Involved Biol. (2015) 4:907–22. 10.3390/biology4040907 26690236 PMC4690022

[37] Torrecillas S Makol A Benítez-Santana T Caballero MJ Montero D Sweetman J. Reduced gut bacterial translocation in European sea bass (*Dicentrarchus labrax*) fed mannan oligosaccharides (MOS). Fish Shellfish Immunol. (2011) 30:674–81. 10.1016/j.fsi.2010.12.020 21195771

[38] Vogt L Ramasamy U Meyer D Pullens G Venema K Faas MM. Immune modulation by different types of β2 → 1-fructans is toll-like receptor dependent. PLoS ONE. (2013) 8:e68367. 10.1371/journal.pone.0068367 23861894 PMC3702581

[39] Smith NC Christian SL Taylor RG Santander J Rise ML. Immune modulatory properties of 6-gingerol and resveratrol in Atlantic salmon macrophages. Mol Immunol. (2018) 95:10–9. 10.1016/j.molimm.2018.01.004 29367081

[40] Szodoray P Nakken B Gaal J Jonsson R Szegedi A Zold E. The complex role of vitamin D in autoimmune diseases. Scand J Immunol. (2008) 68:261–9. 10.1111/j.1365-3083.2008.02127.x 18510590

[41] Chagas C Borges MC Martini L Rogero M. Focus on Vitamin D, Inflammation and Type 2 Diabetes. Nutrients. (2012) 4:52–67. 10.3390/nu4010052 22347618 PMC3277101

[42] Soto-Dávila M Hossain A Chakraborty S Rise ML Santander J. *Aeromonas salmonicida* subsp. salmonicida early infection and immune response of Atlantic cod (*Gadus morhua* L) primary macrophages. Front Immunol. (2019) 10:1237. 10.3389/fimmu.2019.01237 31231379 PMC6559310

[43] Sung K Khan SA Nawaz MS Khan AA. A simple and efficient Triton X-100 boiling and chloroform extraction method of RNA isolation from Gram-positive and Gram-negative bacteria. FEMS Microbiol Lett. (2003) 229:97–101. 10.1016/S0378-1097(03)00791-2 14659548

[44] Santander J Kilbourne J Park JY Martin T Loh A Diaz I. Inflammatory effects of *Edwardsiella ictaluri* lipopolysaccharide modifications in catfish gut. Infect Immun. (2014) 82:3394–404. 10.1128/IAI.01697-14 24866806 PMC4136215

[45] Sambrook J Russell DW Molecular Cloning: A Laboratory Manual. 3 rd ed. Cold Spring Harbor, NY: Cold Spring Harbor Laboratory (2001).

[46] Xue X Hixson SM Hori TS Booman M Parrish CC Anderson DM. Atlantic salmon (*Salmo salar*) liver transcriptome response to diets containing *Camelina sativa* products. Comp Biochem Physiol D Genomics Proteomics. (2015) 14:1–15. 10.1016/j.cbd.2015.01.005 25681993

[47] Pontigo JP Agüero MJ Sanchez P Oyarzún R Vargas-Lagos C Mancilla J. Identification and expressional analysis of NLRC5 inflammasome gene in smolting Atlantic salmon (*Salmo salar*). Fish Shellfish Immunol. (2016) 58:259–65. 10.1016/j.fsi.2016.09.031 27640334

[48] Heidaru Z Tinsley J Bickerdike R McLoughlin MF Zou J Martin SAM Antiviral and metabolic gene expression responses to viral infection in Atlantic salmon (*Salmo salar*). Fish Shellfish Immunol. (2015) 42:297–305. 10.1016/j.fsi.2014.11.003 25462555

[49] Caballero-Solares A Hall JR Xue X Eslamloo K Taylor RG Parrish CC. The dietary replacement of marine ingredients by terrestrial animal and plant alternatives modulates the antiviral immune response of Atlantic salmon (*Salmo salar*). Fish Shellfish Immunol. (2017) 64:24–38. 10.1016/j.fsi.2017.02.040 28242361

[50] Pfaffl MW. A new mathematical model for relative quantification in real-time RT–PCR. Nucleic Acids Res. (2001) 29:e45. 10.1093/nar/29.9.e45 11328886 PMC55695

[51] Livak KJ Schmittgen TD Analysis of relative gene expression data using real-time quantitative PCR and the 2^−Δ*ΔCT*^ method. Methods. (2001) 25:402–8. 10.1006/meth.2001.1262 11846609

[52] Øverland H Pettersen EF Rønneseth A Wergeland HI. Phagocytosis by B-cells and neutrophils in Atlantic salmon (*Salmo salar* L.) and Atlantic cod (*Gadus morhua* L.). Fish Shellfish Immunol. (2010) 28:193–204. 10.1016/j.fsi.2009.10.021 19874896

[53] Pierens SL Fraser DR. The origin and metabolism of vitamin D in rainbow trout. J Steroid Biochem Mol Biol. (2015) 145:58–6. 10.1016/j.jsbmb.2014.10.005 25305412

[54] Holick MF Holick SA Guillard RL On the origin and metabolism of vitamin D in the sea. In: Oguro C Pang PKT, editors. Comparative Endocrinology of Calcium Regulation. Tokyo: Japan Scientific Societies Press (1982). p. 85–91.

[55] Trang HM Cole DE Rubin LA Pierratos A Siu S Vieth R. Evidence that vitamin D3 increases serum 25-hydroxyvitamin D more efficiently than does vitamin D2. Am J Clin Nutr. (1998) 68:854–8. 10.1093/ajcn/68.4.854 9771862

[56] Ostermeyer U Schmidt T Vitamin D and provitamin D in fish. Eur Food Res Technol. (2006) 222:403–13. 10.1007/s00217-005-0086-y

[57] Cipriano RC Bullock G Furunculosis and other diseases caused by Aeromonas salmonicida. Fish disease leaflet 66. Kearneysville, WV: USGS/Leetown Science Center Fish Health Branch (2001).

[58] Connors E Soto-Dávila M Hossain A Vasquez I Gnanagobal H Santander J. Identification and validation of reliable *Aeromonas salmonicida* subspecies salmonicida reference genes for differential gene expression analyses. Infect Genet Evol. (2019) 73:314–21. 10.1016/j.meegid.2019.05.011 31108238

[59] Zhang H Wakisaka N Maeda O Yamamoto T. Vitamin C inhibits the growth of a bacterial risk factor for gastric carcinoma: Helicobacter pylori. Cancer. (1997) 80:1897–903 9366290

[60] Kallio J Jaakkola M Mäki M Kilpeläinen P Virtanen V. Vitamin C inhibits *Staphylococcus aureus* growth and enhances the inhibitory effect of quercetin on growth of Escherichia coli *in vitro*. Planta Med. (2012) 78:1824–30. 10.1055/s-0032-1315388 23059632

[61] Aarestrup FM Scott NL Sordillo LM. Ability of Staphylococcus aureus coagulase genotypes to resist neutrophil bactericidal activity and phagocytosis. Infect Immun. (1994) 62:5679–82. 7960153 10.1128/iai.62.12.5679-5682.1994 PMC303320

[62] Gutiérrez-Barroso A Anaya-Lopez JL Lara-Zárate L Loeza-Lara PD López-Meza JE Ochoa-Zarzosa A. Prolactin stimulates the internalization of *Staphylococcus aureus* and modulates the expression of inflammatory response genes in bovine mammary epithelial cells. Vet Immunol Immunopathol. (2008) 121:113–22. 10.1016/j.vetimm.2007.09.007 17988748

[63] Samuel S Sitrin MD. Vitamin D's role in cell proliferation and differentiation. Nutr Rev. (2008) 66:116–24. 10.1111/j.1753-4887.2008.00094.x 18844838

[64] Jakobsen J Smith C Bysted A Kevin D Cashman 3 vitamin D in wild and farmed Atlantic salmon (*Salmo salar*) – What do we know? Nutrients. (2019) 11:982 10.3390/nu11050982 PMC6566758 31036792

[65] Barnett BJ Cho CY Slinger SJ The essentiality of cholecalciferol in the diets of rainbow trout (*Salmo gairdneri*). Comp Biochem Physiol. (1979) 63:291–7. 10.1016/0300-9629(79)90162-2

[66] Barnett BJ Cho CY Slinger SJ. Relative biopotency of dietary ergocalciferol and cholecalciferol and the role of and requirement for vitamin D in rainbow trout (*Salmo gairdneri*). J Nutr. (1982) 112:2011–9. 6290624 10.1093/jn/112.11.2011

[67] Martin S Blaney S Houlihan D Secombes CJ. Transcriptome response following administration of a live bacterial vaccine in Atlantic salmon (*Salmo salar*). Mol Immunol. (2006) 43:1900–11. 10.1016/j.molimm.2005.10.007 16313960

[68] Bridle A Nosworthy E Polinski M Nowak B. Evidence of an antimicrobial-immunomodulatory role of Atlantic salmon cathelicidins during infection with Yersinia ruckeri. PLoS ONE. (2011) 6:e23417. 10.1371/journal.pone.0023417 21858109 PMC3153500

[69] Santana A Salinas N Álvarez C Mercado L Guzmán F. Alpha-helical domain from IL-8 of salmonids: mechanism of action and identification of a novel antimicrobial function. Biochem Biophys Res Commun. (2018) 498:803–9. 10.1016/j.bbrc.2018.03.061 29530531

[70] Hoare R Jung S Ngo TPH Bartie K Bailey J Thompson KD Adams A. Efficacy and safety of a non-mineral oil adjuvanted injectable vaccine for the protection of Atlantic salmon (*Salmo salar* L.) against Flavobacterium psychrophilum. Fish Shellfish Immun. (2019) 85:44–51. 10.1016/j.fsi.2017.10.005 29017943

[71] Finlay B McFadden G. Anti-immunology: evasion of the host immune system by bacterial and viral pathogens. Cell. (2006) 124:767–82. 10.1016/j.cell.2006.01.034 16497587

[72] Monack D Mecsas J Bouley D Falkow S Yersinia-induced apoptosis in vivo aids in the establishment of a systemic infection of mice. J Exp Med. (1998) 216:2127–37. 10.1084/jem.188.11.2127 PMC2212385 9841926

[73] Schesser K Splik A Dukuzumuremyi J Neurath M Petterson S Wolf-Watz H The *yopJ* locus is required for *Yersinia*-mediated inhibition of NF-kB activation and cytokine expression: YopJ contains a eukaryotic SH2- like domain that is essential for its repressive activity. Mol Microbiol. (1998) 28:1067–79. 10.1046/j.1365-2958.1998.00851.x 9680199

[74] Gao LY Kwaik YA. The modulation of host cell apoptosis by intracellular bacterial pathogens. Trends Microbiol. (2000) 8:306–13. 10.1016/S0966-842X(00)01784-4 10878765

[75] Fast MD Muise DM Easy RE Ross NW Johnson SC. The effects of *Lepeophtheirus salmonis* infections on the stress response and immunological status of Atlantic salmon (*Salmo salar*). Fish Shellfish Immunol. (2006) 21:228–41. 10.1016/j.fsi.2005.11.010 16483797

[76] Lewis DL Barker DE McKinley RS. Modulation of cellular innate immunity by *Lepeophtheirus salmonis* secretory products. Fish Shellfish Immun. (2014) 38:175–83. 10.1016/j.fsi.2014.03.014 24657318

[77] Yamagoe S Yamakawa Y Matsuo Y Minowada J Mizuno S Suzuki K Purification and primary amino acid sequence of a novel neutrophil chemotactic factor LECT2. Immunol Lett. (1996) 51:9–13. 10.1016/0165-2478(96)02572-2 8877413

[78] Rabinovitch M. Professional and non-professional phagocytes: an introduction. Trends Cell Biol. (1995) 5:85–7. 10.1016/S0962-8924(00)88955-2 14732160

[79] Neumann NF Stafford JL Barreda D Ainsworth AJ Belosevic M Antimicrobial mechanisms of fish phagocytes and their role in host defence. Dev Comp Immunol. (2001) 25:807–25. 10.1016/S0145-305X(01)00037-4 11602197

[80] Hardie LJ Fletcher TC Secombes CJ The effect of vitamin E on the immune response of the Atlantic Salmon (*Salmo salar* L.). Aquaculture. (1990) 87:1–13. 10.1016/0044-8486(90)90206-3

[81] Hardie LJ Fletcher TC Secombes CJ The effect of dietary vitamin C on the immune response of the Atlantic salmon (*Salmo salar* L.). Aquaculture. (1991) 95:201–14. 10.1016/0044-8486(91)90087-N

