# Supplementary material for: Effects of Vitamin D2 (Ergocalciferol) and D3 (Cholecalciferol) on Atlantic Salmon (Salmo salar) Primary Macrophage Immune Response to Aeromonas salmonicida subsp. salmonicida Infection
Source: Front Immunol. 2020 Jan 14;10:3011. doi: 10.3389/fimmu.2019.03011 (PMC6973134; doi:10.3389/fimmu.2019.03011)
Supplement: Supplementary file 1 [file Table_1.docx]

**Supplementary Table 1.** Ct values Atlantic salmon housekeeping evaluation.

| Treatment | Fish | *18S* | *Ef-1α* | *60S* | *β-actin* | *HPRT* |
| --- | --- | --- | --- | --- | --- | --- |
| Control (PBS) | **1** | 18.745 | 22.513 | 19.608 | 17.504 | 25.782 |
|  | **2** | 19.938 | 23.454 | 19.522 | 16.836 | 26.496 |
|  | **3** | 18.711 | 22.164 | 19.525 | 17.052 | 26.600 |
| *A. salmonicida* J223 infected cells | **1** | 20.020 | 23.742 | 18.406 | 17.577 | 26.776 |
|  | **2** | 20.204 | 23.953 | 19.414 | 17.303 | 27.077 |
|  | **3** | 19.754 | 21.448 | 19.689 | 17.921 | 26.206 |
| Formalin-killed *A. salmonicida* J223 inoculated cells | **1** | 20.151 | 22.061 | 19.364 | 16.850 | 25.634 |
|  | **2** | 18.658 | 21.836 | 19.979 | 17.541 | 26.155 |
|  | **3** | 19.715 | 24.831 | 18.749 | 17.598 | 24.276 |
| 100 ng/ml vitamin D_2_ pre-treated cells | **1** | 20.331 | 25.450 | 19.083 | 16.844 | 24.892 |
|  | **2** | 17.652 | 25.593 | 19.307 | 16.979 | 26.380 |
|  | **3** | 20.669 | 26.071 | 19.333 | 17.455 | 26.293 |
| 100 ng/ml vitamin D_3_ pre-treated cells | **1** | 19.028 | 24.483 | 18.742 | 17.943 | 25.753 |
|  | **2** | 18.997 | 24.976 | 19.166 | 17.436 | 26.120 |
|  | **3** | 17.681 | 23.565 | 18.900 | 17.578 | 24.148 |
| 1,000 ng/ml vitamin D_2_ pre-treated cells | **1** | 19.758 | 23.819 | 18.795 | 17.805 | 24.662 |
|  | **2** | 19.553 | 25.884 | 18.752 | 17.580 | 26.308 |
|  | **3** | 17.710 | 27.962 | 19.749 | 17.780 | 27.376 |
| 1,000 ng/ml vitamin D_3_ pre-treated cells | **1** | 19.556 | 25.843 | 19.047 | 17.873 | 26.517 |
|  | **2** | 18.875 | 25.702 | 18.648 | 17.460 | 25.931 |
|  | **3** | 18.325 | 19.930 | 18.869 | 17.468 | 27.337 |
| 100 ng/ml vitamin D_2_ pre-treated and *A. salmonicida* J223 infected cells | **1** | 17.344 | 21.842 | 18.411 | 17.662 | 26.637 |
|  | **2** | 17.228 | 21.242 | 18.602 | 17.453 | 25.988 |
|  | **3** | 17.845 | 26.867 | 19.366 | 18.040 | 26.539 |
| 100 ng/ml vitamin D_3_ pre-treated and *A. salmonicida* J223 infected | **1** | 18.711 | 24.814 | 18.710 | 17.621 | 26.033 |
|  | **2** | 18.344 | 23.575 | 19.386 | 18.034 | 26.055 |
|  | **3** | 17.411 | 21.842 | 18.975 | 17.738 | 26.347 |

*****Each value represent the mean of technical replicates (n=3).
******geNorm M values were: 0.592 (60S), 0.592 (β-actin), 0.761 (HPRT), 0.991 (18S), 1.408 (Ef-1α); M<0.15 for most stable genes [1].
*******BestKeeper values were: 0.263 (β-actin), 0.364 (60S), 0.577 (HPRT), 0.865 (18S), 1.604 (Ef-1α); M<1 for most stable genes [2].

1. Vandesompele, J., De Preter, K., Pattyn, F., Poppe, B., Van Roy, N., De Paepe, A., and Speleman, F. (2002). Accurate normalization of real-time quantitative RT-PCR data by geometric averaging of multiple internal control genes. Genome Biol. 3, research0034-research0034.11.
2. Pfaffl, M., Tichopad, A., Prgomet, C., and Neuvians, T.P. (2004). Determination of stable housekeeping genes, differentially regulated target genes and sample integrity: BestKeeper – Excel-based tool using pair-wise correlations. Biotechnol. Lett. 26, 509-515.
