# Supplementary material for: Effects of Vitamin D2 (Ergocalciferol) and D3 (Cholecalciferol) on Atlantic Salmon (Salmo salar) Primary Macrophage Immune Response to Aeromonas salmonicida subsp. salmonicida Infection
Source: Front Immunol. 2020 Jan 14;10:3011. doi: 10.3389/fimmu.2019.03011 (PMC6973134; doi:10.3389/fimmu.2019.03011)
Supplement: Supplementary file 2 [file Image_1.pdf]

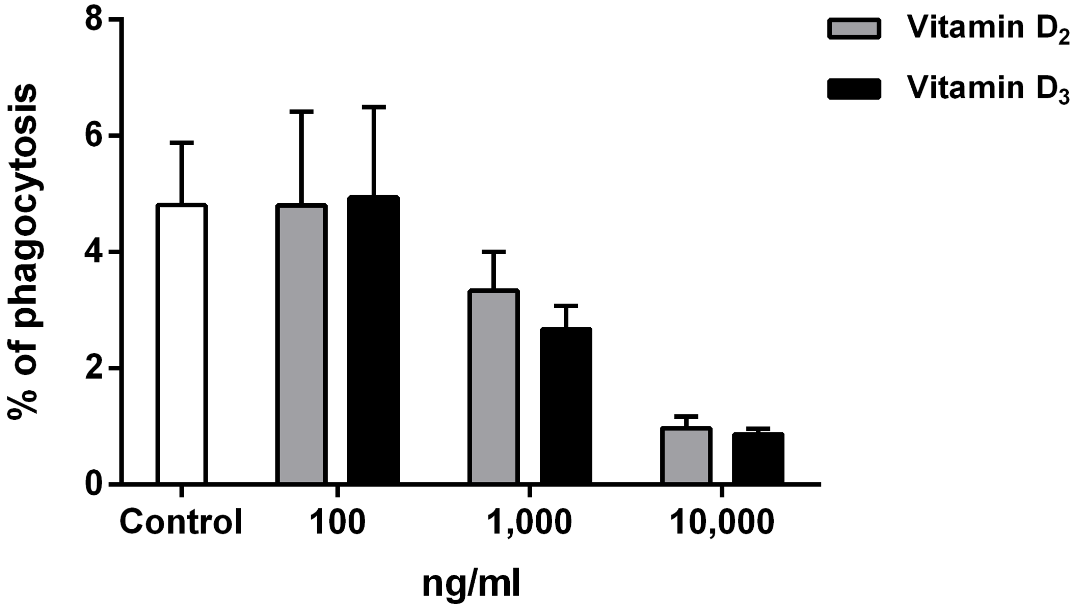

Supplementary Figure 1. Effect of vitamin D_2 and vitamin D_3 in Atlantic salmon primary macrophage phagocytosis. Macrophages were pre-treated with 100, 1,000, and 10,000 ng/ml of vitamin D_2 or vitamin D_3 for 24 h, and then inoculated with 1 µm of Fluoresbrite YG microspheres. A non pre-treated control was utilized to determine the percentage of FITC-positive positive cells. Phagocytosis was determined by flow cytometry. Each value represents the mean ± S.E.M (n = 3), $p < 0.05$.
